# Are medication-induced salivary changes the culprit of osteonecrosis of the jaw? A systematic review

**DOI:** 10.3389/fmed.2023.1164051

**Published:** 2023-08-31

**Authors:** Isti Rahayu Suryani, Iraj Ahmadzai, Minh Ton That, Sohaib Shujaat, Reinhilde Jacobs

**Affiliations:** ^1^OMFS IMPATH Research Group, Department of Oral and Maxillofacial Surgery and Imaging and Pathology, Faculty of Medicine, University Hospitals Leuven, KU Leuven, Leuven, Belgium; ^2^Department of Dentomaxillofacial Radiology, Faculty of Dentistry, Universitas Gadjah Mada, Yogyakarta, Indonesia; ^3^King Abdullah International Medical Research Center, Department of Maxillofacial Surgery and Diagnostic Sciences, College of Dentistry, Ministry of National Guard Health Affairs, King Saud bin Abdulaziz University for Health Sciences, Riyadh, Saudi Arabia; ^4^Department of Dental Medicine, Karolinska Institute, Stockholm, Sweden

**Keywords:** polypharmacy, saliva, xerostomia, adverse drug reaction, osteonecrosis of the jaw

## Abstract

**Purpose:**

This systematic review was performed to assess the potential influence of medication-induced salivary changes on the development of medication-related osteonecrosis of the jaw (MRONJ).

**Methods:**

An electronic search was conducted using PubMed, Web of Science, Cochrane, and Embase databases for articles published up to June 2023. A risk of bias assessment was performed according to the modified Newcastle–Ottawa Scale (NOS). Due to the heterogeneity of the selected studies in relation to the type of medications and outcomes evaluated, a meta-analysis could not be performed.

**Results:**

The initial search revealed 765 studies. Only 10 articles were found to be eligible based on the inclusion criteria that reported on the impact of salivary changes on MRONJ following the administration of different medications. A total of 272 cases of MRONJ (35% women, 32% men, and 32% with no gender reported) with a mean age of 66 years at the time of diagnosis were included. Patients administered with bisphosphonates, steroids, chemotherapy, thalidomide, interferon, and hormone therapy had a significantly higher association between decreased salivary flow and MRONJ occurrence. In addition, bisphosphonates, denosumab, and other bone-modifying agents showed a significantly higher risk of developing MRONJ owing to the changes in salivary microbiome profiles, cytokine profiles, interleukins, hypotaurine, and binding proteins.

**Conclusion:**

The reduction in salivary flow and changes in the concentration of salivary proteins were associated with the development of MRONJ. However, due to the availability of limited evidence, the findings of the review should be interpreted with caution.

**Prospero review registration:**

https://www.crd.york.ac.uk/PROSPERO/, identifier: CRD42022327645.

## Introduction

Medication-related osteonecrosis of the jaw (MRONJ) is an adverse drug reaction, described as an exposed necrotic bone or a bone that can be probed through an intraoral or extraoral fistula in the maxillofacial region, that persists for more than 8 weeks in patients without a history of radiotherapy or disease metastasis to the jaws (1, 2). It commonly occurs in oncology patients receiving pharmacological agents, such as antiresorptive drugs, antiangiogenic drugs, immunomodulators, and immunosuppressants (3, 4). The pathological mechanism of MRONJ varies depending on the administered drugs. However, the main mechanism of the majority of drugs involves impairment of bone remodeling via the inhibition of osteoclastic activity, the induction of cell apoptosis, and/or the disruption of blood vessel formation through the deterioration of vascular endothelial growth factor. The frequency of MRONJ is highest in patients with multiple myeloma, and its occurrence rate is more in osteoporotic patients compared with the general population (5, 6).

The early imaging signs of MRONJ include bone sclerosis, lamina dura thickening, alveolar socket persistence following tooth extraction, periapical radiolucency, robust mandibular cortex, expanded periodontal ligament space, receding periodontal bone, and an expanded mandibular canal (7–9). These patients often exhibit common symptoms such as pain, infection with purulent discharge, jaw discomfort, paresthesia, malodor, and non-healing extraction site (10). The MRONJ lesions are divided into three stages (stage 0–stage 3) based on clinical and radiological features as proposed by the American Association of Oral and Maxillofacial Surgeons (AAOMS): stage 0: no exposed bone + non-specific signs/symptoms; stage 1: asymptomatic exposed bone; stage 2: symptomatic exposed bone + infection/pain; and stage 3: symptomatic exposed bone + infection + pathological fracture/extraoral fistula/oro-antral or oro-nasal communication/osteolysis extending to inferior mandibular border or sinus floor (11). Their management ranges from conservative therapy with antibiotics, antimicrobials, and analgesics to surgical debridement or sequestrectomy, depending on disease severity. To prevent MRONJ occurrence, it is important to appropriately maintain the oral hygiene of the patient, treat oral infections, and complete all dental surgical procedures before initiating osteonecrosis of the jaw-related medications. Moreover, during drug therapy, patients should undergo regular dental screening to prevent possible future occurrences (12).

Current evidence indicates that MRONJ is a multifactorial consequence arising from the direct periodontal tissue infection (13–15), distinctive oral microflora or biofilm (16), invasive oral surgical procedures (17, 18), systemic risk comorbidities (19), and alteration of the local immune system (20). Despite the availability of robust data related to the risk factors for developing MRONJ, the pathogenesis of the disease in relation to changes in salivary mediators is still not well understood.

Saliva plays a vital role in maintaining oral homeostasis due to its protective and functional properties. Some of these include teeth remineralization, buffering and neutralizing intrinsic and extrinsic acids, inhibiting harmful microorganisms' overgrowth, preventing xerostomia, and facilitating speech and swallowing. Any change in the salivary function would lead to a plethora of complications and result in a decreased quality of life (21, 22). It is a known fact that the medications responsible for MRONJ are also responsible for altering salivary composition, levels, and secretion. These salivary dysfunctions have significantly been associated with a higher incidence of dental caries. However, few studies have assessed the association between salivary changes and MRONJ occurrence. To the best of our knowledge, no previous systematic approach has been applied to investigate the relationship between salivary changes and MRONJ. Therefore, this review aimed to explore the link between medication-related salivary changes and the development of MRONJ.

## Materials and methods

### Protocol and registration

The study protocol was registered in the PROSPERO database under the number CRD42022327645. The systematic review followed the Preferred Reporting Items for Systematic Reviews and Meta-Analyses (PRISMA) statement with 23 items (23). The review question was formulated according to the patient, intervention, comparison, and outcomes (PICO) framework as follows:

P: cancer and osteoporosis

I: medications that induce salivary changes

C: no comparators

O: MRONJ

“What is the association between the use of medications that induce salivary changes (I) and the occurrence of MRONJ (O) among cancer and osteoporotic patients (P)?”

### Search strategy

An electronic literature search was conducted using PubMed (https://www.pubmed.ncbi.nlm.nih.gov), Web of Science (https://www.webofscience.com), Cochrane (https://www.cochranelibrary.com), and Embase (https://www.embase.com) databases, from January 2013 to June 2023. The search was restricted in the past 10 years with a goal to include the most recent body of evidence related to drug administration in oncology and osteoporotic patients. Studies evaluating the possible association between salivary changes due to medications and MRONJ occurrence were identified. The search was conducted using the following MeSH terms, topics, and keywords: “hyposalivation”, “xerostomia”, “dry mouth”, “burning mouth syndrome”, “mouth dryness”, “osteonecrosis of the jaw”, “osteonecrosis/drug therapy”, “medication-related osteonecrosis of the jaw”, “MRONJ”, “BRONJ”, “ARONJ”, and “human”. No language restriction was applied. The complete search strategy is provided in Appendix 1. A gray literature search was performed on ProQuest, OpenGrey, and Google Scholar, followed by a manual search of cross-references within the selected studies.

### Eligibility criteria

The full text of relevant articles was acquired based on the inclusion and exclusion criteria. The inclusion study consisted of both children and adult human clinical studies that assessed medication-related salivary changes and osteonecrosis of the jaw occurrence. The exclusion criteria were animal studies, *in vitro* studies, case reports, systematic reviews, conference abstracts, letters, editorials, and surveys. In addition, studies involving patients with a history of radiotherapy, disease metastasis to the jaws, and surgical intervention of head and neck cancer impacting the salivary flow were also excluded.

### Study selection

The identified articles were imported into EndNote X9 software (Thomson Reuters, Philadelphia, PA, USA). Following the removal of duplicates, two independent reviewers (IA and MT) screened the articles based on the titles and abstracts. Subsequently, the full text of the articles deemed eligible for inclusion was obtained. Reasons for exclusion were also recorded. Any disagreement between reviewers was resolved through discussion, and a third expert (IS) was consulted if a consensus could not be reached. The Cohen's kappa coefficient was employed to assess the agreement between the reviewers for the selection process.

### Data extraction

The extracted data included the following: title, author, year of publication, study design, number of patients, gender, age, underlying disease, pharmacological agents, and type of salivary changes.

### Risk of bias assessment

The risk of bias (RoB) was assessed with the Newcastle–Ottawa Scale (NOS) (24) by two independent reviewers (IA and MT). The NOS tool was adapted to assess the selection (maximum 4 stars), comparability (maximum 2 stars), and outcome (maximum 3 stars) parameters, with a total score of 9 stars. The study quality was categorized as good, fair, or poor, based on the modified NOS guidelines.

## Results

### Study selection

**Figure 1:**
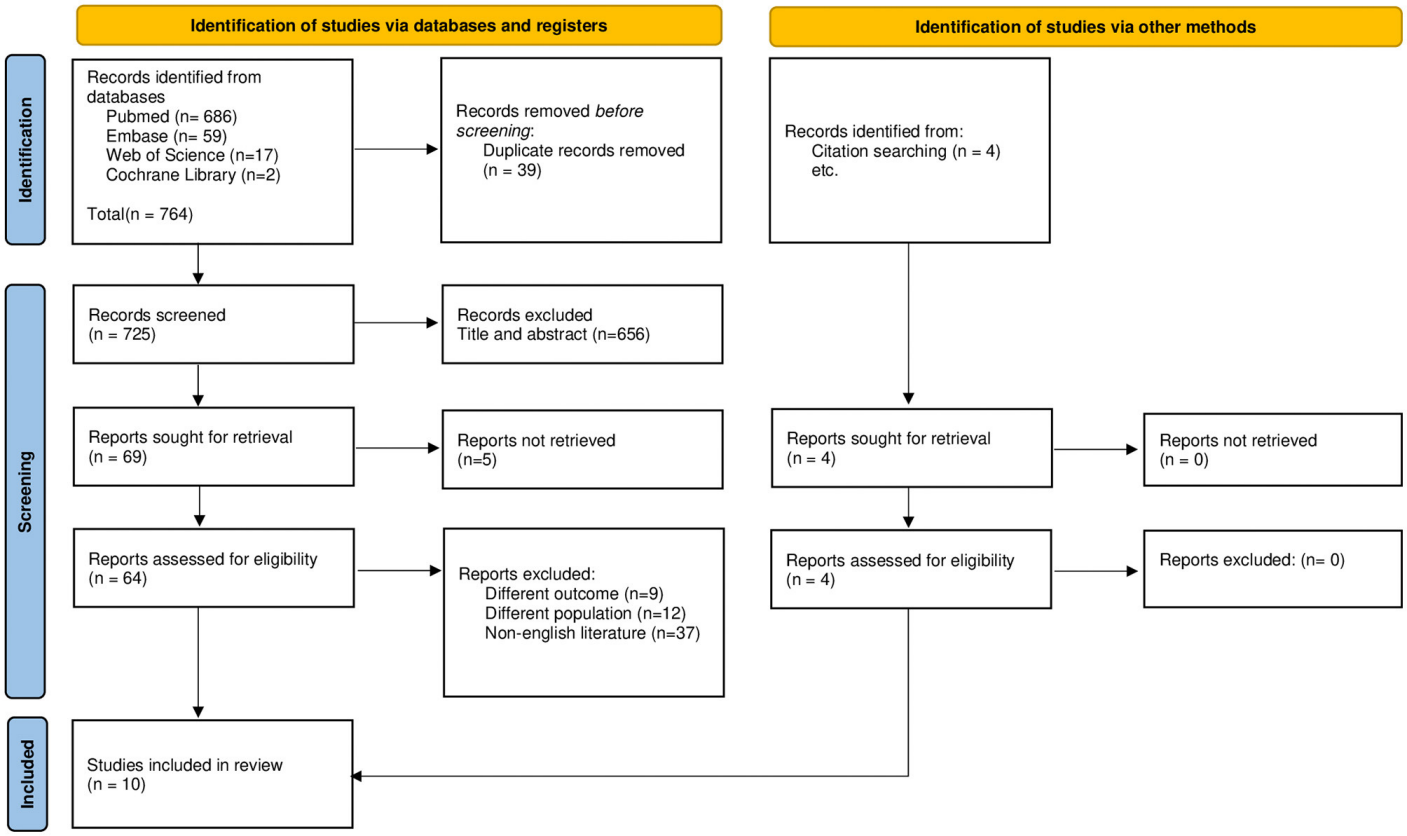
PRISMA flowchart.

Figure 1 illustrates the flowchart of the entire selection process based on the PRISMA guidelines. The search strategy yielded a total of 765 articles. Following the removal of duplicates, title and abstract screening, and full-text reading, 10 studies were found to be eligible based on the selection criteria to be included in the qualitative synthesis. Quantitative synthesis was not possible owing to the heterogeneity in assessment methodologies, pharmacological agents, and reported outcomes.

### Study characteristics

Table 1 presents the summary of the patients and disease characteristics. A total of 272 cases of MRONJ (35% women, 32% men, and 32% with no gender reported) with a mean age of 66 years (range: 33–81 years) at the time of diagnosis were included. The predominant primary disease and comorbid conditions reported were breast cancer (*n* = 57) and hypertension (*n* = 107), respectively. The most common salivary change was xerostomia (dry mouth) due to Sjogren's syndrome (SS = 35), and the main pharmacological contributors for MRONJ occurrence were bisphosphonates (*n* = 530), followed by chemotherapy (*n* = 11) and corticosteroids (*n* = 11).

**Table 1:** Summary of patients and disease characteristics.

**Reported cases MRONJ**	***n =* 272**	**100%**
**Gender**		
Women	96	35.29
Men	88	32.35
Not reported	88	32.35
**Age**		
Mean	65.6	
Min	33	
Max	81	
**Primary disease**		
Multiple myeloma	55	20.22
Breast cancer	57	20.95
Prostate cancer	19	6.98
Renal cancer	8	2.94
Other Cancer	58	21.32
Osteoporosis	19	6.98
**Comorbid conditions**		
Diabetes mellitus	30	11.02
Hypertension	107	37.5
**Medications**		
Bisphosphonate	530	85.76
Denosumab	4	0.65
Corticosteroids	11	1.78
Chemotherapy	11	1.78
Others	14	2.27

MRONJ, medication-related osteonecrosis of the jaw.

### Qualitative synthesis

The characteristics of the 10 included studies and the significance of the association between salivary alterations by pharmacological agents and MRONJ occurrence are presented in Table 2. The included study designs were case–control (*n* = 6), cohort (*n* = 3), and retrospective (*n* = 1) in nature.

**Table 2:** Characteristics of the included studies in correlation to MRONJ.

**References**	**Location**	**Study design**	**Age (mean)**	**n**	**MRONJ cases**	**Gender**	**Underlying conditions**	**Medications**	**Sialometric assessment**	**Saliva changes**	***P*-value**
Liao et al. (25)	Taiwan	Retrospective cohort	57.4	13,398	11	nm	Malignancy	Bisphosphonates	nm	Saliva flow↓	0.017^*^
							Diabetes mellitus	Steroids			
							Hypertension	Chemotherapy			
							Chronic Kidney Disease				
							Osteoporosis				
Margaix-Muñoz et al. (26)	Spain	Case-control	63.7	156	67	39 F, 28 M	Multiple Myeloma	Bisphosphonates	RWS and SWS	Resting whole saliva↓	>0.05
							Breast Cancer	Corticosteroids			
							Prostate Cancer	Thalidomide			
							Lung Cancer	Interferon			
							Kidney Cancer	Hormones			
							Bladder Cancer			Stimulated whole saliva↓	>0.05
Kuo et al. (27)	Taiwan	Retrospective	71.05	339	19	nm	Osteoporosis	Bisphosphonates	nm	Saliva flow↓	0.0024^*^
							Sjörgren's syndrome	Non-bisphosphonates			
							Diabetes mellitus				
							Dyslipidemia				
							Hypothyroidism				
							Hyperthyroidism				
							Anemia				
							Chronic Kidney Disease				
							Esophagitis or ulcers				
							Peptic ulcers				
Badros et al. (28)	USA	Observational prospective	60	110	14	5F, 9M	Multiple Myeloma	Bisphosphonates	RWS every 3 months	MIP-1β↑	0.01^*^
							Diabetes mellitus	Lenalidomide		TNF-α↑	0.09
							Smoking	Carfilzomib		IL-6↑	0.02^*^
								Other			
Lorenzo-Pouso et al. (29)	Spain	Case-control	69.8	586	18	14 F, 4 M	Breast Cancer	IV zoledronate	RWS	MMP9↑	< 0.05
							Osteoporosis	Oral pamidronate			
							Multiple Myeloma	Subcutaneous denosumab			
							Prostate Cancer	BMA			
							Others				
Stockmann et al. (30)	Germany	Case-control	70	60	20	9 F, 11 M	Breast cancer	Bisphosphonates	SWS	Saliva flow↓	0.039
							Prostate cancer				
							Multiple myeloma				
							Cervix cancer				
							Osteoporosis				
Bagan et al. (31)	Spain	Case-control	66.1	70	30	15 F, 15 M	Breast cancer	IV Bisphosphonates	RWS	IL-6↑	< 0.01
							Multiple myeloma				
							Prostate cancer				
							Lungs cancer				
							Kidney cancer				
							Sarcoma				
Bagan et al. (32)	Spain	Case-control	65.7	81	26	10 F, 16 M	Breast cancer	Bisphosphonates	RWS	IL-1α↑	< 0.05
							Multiple myeloma			IL-1β↑	< 0.05
							Prostate cancer			IL-IRA↑	< 0.05
							Renal cancer				
Yatsuoka et al. (33)	Japan	Cohort	70.8	35	9	4 F, 5 M	Solid cancer	BMA	Metabolic analysis	Hypotaurine↑	0.017
Nicoletti et al. (34)	USA	Case-control	62.8	67	53	nm	nm	Bisphosphonates	Questionnaire and genotyping	RMBS3↑	< 7 × 10^8^

F, female; M, male; nm, not mentioned; MRONJ, medication-related osteonecrosis of the jaw; IV, intravenous; BMA, bone modifying agents; IL, interleukin; IL-IRA, interleukin-1 receptor antagonist; MMP9, matrix metallopeptidase 9; RMBS3, ribonucleic acid binding motif single stranded interacting protein 3; MIP, macrophage inflammatory protein; TNF, tumor necrosis factor; α, alpha; β, beta; RWS, resting whole saliva; SWS, stimulated whole saliva. The ^*^ symbol indicates the statistical significance.

Based on the qualitative synthesis, a reduction in salivary flow and changes in salivary protein levels led to the development of MRONJ. Two studies revealed a significant association between SS and MRONJ, following the administration of bisphosphonates, steroids, and chemotherapy (25, 27). In two studies, patients with a history of bisphosphonates, steroids, chemotherapy, thalidomide, interferon, and hormone therapy showed a significantly higher association between salivary flow and MRONJ development (26, 30). Moreover, bisphosphonates, denosumab, and other bone-modifying agents were associated with a significantly higher risk of developing MRONJ owing to the changes in the microstructure of saliva in relation to microbiome profiles, cytokine profiles, interleukins (ILs), hypotaurine, RNA-binding motifs, and the single-stranded-interacting protein 3 (RMBS3) gene (28, 29, 31–34).

### Within-study risk of bias

**Figure 2:**
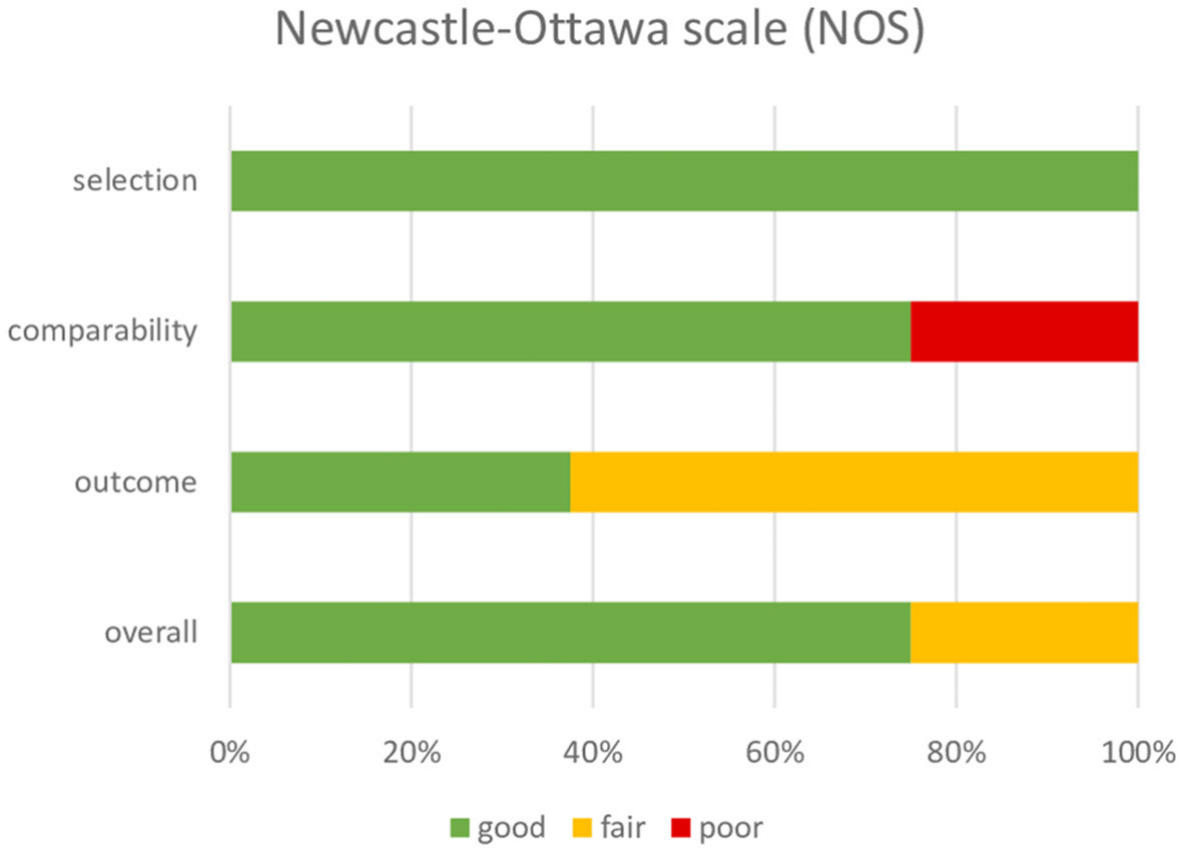
Rating Newcastle–Ottawa Scale (NOS).

Based on the modified NOS tool, all studies were rated as good, apart from the one that had a poor-quality rating. Table 3 and Figure 2 describe the quality analysis of included studies per domain. The outcome assessment revealed significant shortcomings; however, overall, the studies had good (78%) and fair quality (22%).

**Table 3:** Result from the Newcastle–Ottawa risk assessment for observational studies.

**References**	**Newcastle–Ottawa Scale**		
	**Selection (max 4)**	**Comparability (max 2)**	**Outcome (max 3)**
Liao et al. (25)	^***^	^**^	^***^
Margaix-Muñoz et al. (26)	^***^	^**^	^***^
Kuo et al. (27)	^***^	^**^	^**^
Badros et al. (28)	^***^	^**^	^***^
Lorenzo-Pouse et al. (29)	^***^	^**^	^**^
Stockman et al. (30)	^****^	^**^	^**^
Bagan et al. (31)	^***^	^**^	^**^
Bagan et al. (32)	^***^	^**^	^**^
Yatsuoka et al. (33)	^****^	^**^	^**^
Nicoletti et al. (34)	^***^	^**^	^**^

^**^, 2 stars; ^***^, 3 stars; ^****^, 4 stars.

## Discussion

In recent years, it has become evident that the changes in salivary properties and constituents are altered in response to various medications and diseases. With the recent advancements in salivaomics, a wide range of discriminatory and definitively validated salivary biomarkers have been established for diagnostic purposes. As a non-invasive and safe source, saliva could replace blood as a medium of choice for diagnosis and for assessing disease prognosis (35); hence, the following review was conducted, which might enable the isolation of certain salivary factors for a better understanding of the pathophysiology of MRONJ.

A majority of the included studies diagnosed changes in salivary flow by collecting samples of either resting or stimulated whole saliva, while others used metabolic analysis and genotyping. The lack of standardization in saliva collection techniques, targeted biomarkers, and analytical methods precluded a direct comparison of data across studies. A higher percentage of patients with MRONJ were receiving chemotherapy at the time of saliva collection compared with non-MRONJ patients, which may partially explain the observed differences in oral health (36). The prolonged use of bisphosphonates and concurrent chemotherapy in MRONJ patients might also contribute to oral disorders, not only through reduced salivary gland function but also through increased susceptibility to fungal and bacterial infections and changes in oral microflora (37, 38). Further research is needed to explore the link between these infections and the development of MRONJ (39).

Based on the findings of the review, a decreased production of saliva was considered to be a risk factor for the development of MRONJ, where xerostomia was mainly triggered by SS following the administration of bone-modifying drugs. In patients with SS, the prevalence of dental caries and early tooth loss is twice as higher and the risk of infection due to *Candida albicans* is 10 times more compared to the general population. All these factors increase the risk of MRONJ, and the health-related quality of life of these patients is severely diminished. It should be noted that patients with SS were more susceptible to bisphosphonate-related osteonecrosis of the jaw, which might be due to the shared risk factors and molecular pathways (25, 27). Low salivary flow in combination with acid reflux might result in an oral environment of low pH, which promotes the growth of acidophilic bacteria, in turn, leading to tooth destruction and mucosal degradation (40). Even in low dosages, these drugs can exert a considerable influence on the expression of genes that play a role in the differentiation and growth of osteoblasts. Potent bisphosphonates, such as zoledronate, have the ability to limit ischemia-induced neovascularization by inhibiting the mobilization of endothelial progenitor cells and angiogenic activities, while zoledronate can reduce bone mineralization within the tooth extraction socket, which causes poor bone healing (25). These outcomes imply that early detection of the changes in salivary flow could act as a diagnostic aid for avoiding MRONJ occurrence and formulating strategies to overcome salivary flow dysfunction.

Polypharmacy was found to be a significant predictor for xerostomia, regardless of the age or gender of the patient. The highest prevalence of xerostomia and MRONJ was observed in patients taking five or more drugs (71%) (41), where xerostomia was determined to be a side effect in approximately 80–100% of the patients. Based on case–control studies, xerostomia and MRONJ occurrence were nearly three times higher compared with patients taking no medication. The mechanism of xerostomia varies depending on the administered drugs. For instance, cytotoxic drugs cause dry mouth by directly damaging the salivary gland, anticholinergic drugs act by interrupting the neural simulation of salivary secretions, and diuretics promote dehydration and excretion of bodily fluids (42). In addition, the majority of medications also decrease the salivary flow by vasoconstriction in the glands (43). Therefore, it is important to identify the dose and type of administered polypharmacy in an attempt to predict the impact of each drug separately as such preventive measures could be taken to lower the risk of oral manifestations and complications.

Recent research has indicated an association between diabetes and decreased saliva production. Chronic high blood sugar levels can negatively impact the salivary glands, where parasympathetic vasodilation and salivary secretion might get impaired. The incidence of hyposalivation has also been shown to be higher in diabetic patients compared with the non-diabetic population. This decrease in salivary flow may further increase the risk of developing MRONJ. In a study examining hyperglycemic patients, the risk of developing MRONJ was significantly higher compared with patients with normal glucose levels. This study analyzed baseline characteristics such as age, gender, cancer type, presence of osteoporosis, and habits, in addition to the possible synergistic impact of hyperglycemia and AR therapy that may result in hyposalivation and ischemia. Ischemia is a potential risk factor for mandibular necrosis following invasive dental or oral surgical procedures (44). Additionally, several factors have been identified to play a role in the pathogenesis of MRONJ in diabetic patients, including compromised bone microenvironment, altered immune cell function, increased infection and inflammation, inhibition of osteoclast function, and induction of apoptosis, microvascular damage, and genetic predisposition (45). Consequently, it is suggested that further research needs to be conducted to accurately evaluate the coexisting medical conditions of patients and their impact on salivary changes, with the aim of developing potential preventative strategies to reduce the incidence of MRONJ.

In relation to bone-modifying agents, the review also suggested that a significant association existed between bisphosphonates, hyposalivation, and MRONJ occurrence. It is unclear whether discontinuation of osteonecrosis of the jaw-related medications would be effective in reducing or preventing MRONJ. The risk of MRONJ varies depending on the type of drug, the frequency of administration, and dose and duration of treatment, where patients treated with high-dose drugs are at a greater risk. A study by Kim et al. proposed that a temporary cessation of bone-modifying drugs, known as a drug holiday, during tooth extraction may reduce the risk of MRONJ (46). However, another study found no evidence to support an association between drug holiday and a reduced risk of MRONJ (47). An alternative solution is to lower the cumulative doses of medications, as suggested by the American Dental Association Council on Scientific Affairs (48). However, to date, no detailed guidelines exist related to the impact of different drugs and alternative therapies to reduce the risk of MRONJ. Hence, future prospective studies are required to reach firmer conclusions.

The findings of the review also demonstrated that changes in salivary proteins acted as a risk factor for the development of MRONJ. Bone-modifying drugs, especially bisphosphonates, increased the production of IL-6 and osteoprotegerin while simultaneously reducing the production of receptor activators of nuclear factor kappa-B ligand (RANKL) (49). This increasing ratio of RANKL to osteoprotegerin signifies that IL-6 is responsible for stimulating osteoclast activity. In addition, IL-6 release is also increased due to the decrease in the enzyme hydroxymethylglutaryl coenzyme A (HMG-CoA), following drug administration. All these changes in the salivary proteins result in a higher occurrence of MRONJ. Bagan et al. suggested that patients with high salivary IL-6 levels following bisphosphonate therapy had a 1.01 odds ratio of developing MRONJ, which increased with the severity of the disease (31, 32). However, Badros et al. proposed that cytokine response was the main culprit in the pathogenesis of MRONJ since the tissue injury in MRONJ patients was associated with a pro-inflammatory cytokine profile indicative of macrophage activation (28).

Other cytokines, such as tumor necrosis factor-alpha (TNF-α) (50, 51) and interleukin-1 beta (IL-1β) (52), might also cause osteonecrosis of the jaw by promoting inflammation and bone resorption. TNF-α together with RANKL, both of which are members of the TNF superfamily, maintain immune homeostasis and contribute toward bone degradation. TNF-α is an osteoclast-stimulating molecule that stimulates osteoclastogenesis either by acting on osteoclast precursors or increasing the production of RANKL. As TNF-α influences bone metabolism similar to RANKL, its inhibition might result in a decrease in bone turnover, following the administration of bone-modifying drugs and lead to the development of MRONJ (53). In addition, IL-1β has also been regarded as potent pro-inflammatory cytokines for stimulating bone resorption by causing the upregulation of RANKL, which ultimately leads to an imbalance in bone metabolism through osteoclastogenesis (54). In relation to MRONJ, it acts as a pro-inflammatory factor and causes delayed wound healing (55).

The findings also suggested that overexpression of matrix metalloproteinases (MMPs), specifically MMP8 and MMP9, was observed in patients with MRONJ when compared with healthy patients. These salivary proteins are collagen-degrading zinc-dependent endopeptidases, primarily produced by macrophages and granulocytes (56), where MMP8 is associated with cancer and MMP9 is related to RANKL expression. In addition, hypotaurine, a cystamine analog and a precursor for taurine synthesis, was also elevated in MRONJ patients. The concentration of taurine in saliva rises as a biological response to bacterial inflammation and infection during MRONJ development. Yatsuoka et al. (33) suggested that its high concentration could enable the detection of MRONJ at an early stage. In relation to genomic profiling, RBMS3 was also significantly associated with MRONJ occurrence. It binds to Prx1, a homeobox transcriptional factor, that increases the expression of collagen type I in fibroblasts (57). The changes in RBMS3 commonly occur following the administration of bisphosphonates, which causes loss of bone mass and osteoporotic fractures. In relation to the diagnostic impact of saliva-based protein biomarkers for identifying patients who are at risk of developing MRONJ and monitoring the progression of the disease, further longitudinal and large-sample-sized studies are required to determine the sensitivity and specificity of each biomarker and establish their predictive value in MRONJ occurrence. The main strength of this systematic review is the inclusion of studies evaluating the association between salivary changes and MRONJ occurrence, which has not been previously investigated. The introduction of clear-cut diagnostic and prediction criteria of MRONJ based on polypharmacy, salivary flow, and biomarkers could act as a step forward in devising patient-specific management guidelines. The review also has certain limitations. First, a limited number of studies, mostly having a small sample size, assessed salivary changes. Second, heterogeneity existed in relation to study design, type of administered drugs, primary disease, and outcome assessment methodologies. Hence, future standardized case–control studies involving a larger cohort of patients are required to identify and confirm the potential association between MRONJ and salivary changes, following the administration of different drug categories.

## Conclusion

The reduction in salivary flow and changes in the concentration of salivary proteins were associated with the development of MRONJ. However, due to the availability of limited evidence, the findings of the review should be interpreted with caution. It is recommended to assess salivary specimens in patients before and after the development of MRONJ to provide a better understanding of the disease and validate biomarkers for early detection of the disease.

## Data availability statement

The original contributions presented in the study are included in the article/Supplementary material, further inquiries can be directed to the corresponding author.

## Author contributions

All authors listed have made a substantial, direct, and intellectual contribution to the work and approved it for publication.
