# Supplementary material for: Are medication-induced salivary changes the culprit of osteonecrosis of the jaw? A systematic review
Source: Front Med (Lausanne). 2023 Aug 31;10:1164051. doi: 10.3389/fmed.2023.1164051 (PMC10501800; doi:10.3389/fmed.2023.1164051)
Supplement: Supplementary file 1 [file Data_Sheet_1.PDF]

Search strategy

Pubmed : 686

((("saliva"[MeSH Terms] OR "saliva"[All Fields] OR "salivas"[All Fields] OR "saliva s"[All Fields] OR ("xerostomia"[MeSH Terms] OR "xerostomia"[All Fields] OR "xerostomias"[All Fields] OR ("hyposalivators"[All Fields] OR "xerostomia"[MeSH Terms] OR "xerostomia"[All Fields] OR "hyposalivation"[All Fields]) OR "dry mouth"[All Fields] OR ("xerostomia"[MeSH Terms] OR "xerostomia"[All Fields] OR "asialia"[All Fields]) OR "burning mouth syndrome"[All Fields] OR "mouth dryness"[All Fields])) AND ("osteonecrosis of the jaw"[Title/Abstract] OR "osteonecrosis/drug therapy"[MeSH Terms] OR "medication related osteonecrosis of the jaw"[Title/Abstract] OR "MRONJ"[Title/Abstract] OR "bisphosphonate associated osteonecrosis of the jaw"[MeSH Terms] OR "BRONJ"[Title/Abstract] OR "ARONJ"[Title/Abstract])) OR "ONJ"[Title/Abstract]) AND ("human s"[All Fields] OR "humans"[MeSH Terms] OR "humans"[All Fields] OR "human"[All Fields])

Embase : 59

('human'/exp OR 'adult'/exp OR 'adult' OR 'adults' OR 'grown-ups' OR 'grownup' OR 'grownups' OR 'child'/exp OR 'child' OR 'children') AND ('saliva'/exp OR 'saliva' OR 'spittle' OR 'salivary gland'/exp OR 'glandula salivaris' OR 'saliva gland' OR 'salivary gland' OR 'salivary glands' OR 'xerostomia'/exp OR 'dry mouth' OR 'mouth dryness' OR 'oral dryness' OR 'xerostomia' OR 'xerostomy' OR 'zerostomiasis' OR 'hyposalivation'/exp OR 'hyposalivation' OR 'hyposialia' OR 'salivation, hypo' OR 'sialoadenitis'/exp OR 'saliva gland inflammation' OR 'salivary gland inflammation' OR 'sialadenitis' OR 'sialoadenitis' OR 'sjoegren syndrome'/exp OR 'gougerot sjoegren syndrome' OR 'gougerot sjogren syndrome' OR 'gougerot-sjogren syndrome' OR 'sjoegren syndrome' OR 'dacryosialoadenopathia atrophicans' OR 'dyssecretosis, mucoserous' OR 'gougerot houwer sjoegren syndrome' OR 'gougerot mulock houwer sjoegren syndrome' OR 'gougerot sjoegren disease' OR 'gougerot sjogren disease' OR 'mikulicz gougerot sjoegren syndrome' OR 'mikulicz radecki syndrome' OR 'mucoserous dyssecretosis' OR 'mukilicz radecki syndrome' OR 'oculobuccopharyngeal dryness' OR 'rheumatic sialosis' OR 'sialosis, rheumatic' OR 'sicca syndrome' OR 'sjoegren disease' OR 'sjogren disease' OR 'sjogren syndrome' OR 'sjogren`s syndrome') AND ('bisphosphonate related osteonecrosis of the jaw'/exp OR 'bonj' OR 'bp-onj' OR 'bp-associated osteonecrosis of the jaw' OR 'bp-induced osteonecrosis of the jaw' OR 'bp-related jaw osteonecrosis' OR 'bp-related osteonecrosis of the jaw' OR 'bponj' OR 'bps osteonecrosis of the jaw' OR 'bps-induced osteonecrosis of the jaw' OR 'bps-related osteonecrosis of the jaw' OR 'bronj' OR 'nbp-associated osteonecrosis of the jaw' OR 'za osteonecrosis of the jaw' OR 'za-onj' OR 'za-induced onj' OR 'za-related osteonecrosis of the jaw' OR 'aminobisphosphonate-associated osteonecrosis of the jaw' OR 'biphosphonate maxillo-mandibular osteonecrosis' OR 'biphosphonate osteonecrosis of the jaw' OR 'biphosphonate-associated osteonecrosis of the mandible' OR 'biphosphonate-associated osteonecrosis of the maxilla' OR 'biphosphonate-associated osteonecrosis of the maxillary bone' OR 'biphosphonate-induced onj' OR 'biphosphonate-related jaw osteonecrosis' OR 'biphosphonate-related osteonecrosis of the jaw' OR 'bisphosphonate associated jaw bone necrosis' OR 'bisphosphonate associated jaw osteonecrosis' OR 'bisphosphonate associated osteonecrosis of the jaw' OR 'bisphosphonate jaw osteonecrosis' OR 'bisphosphonate osteo-necrosis of the jaw' OR 'bisphosphonate osteonecrosis of jaw' OR 'bisphosphonate osteonecrosis of the maxilla' OR 'bisphosphonate related osteonecrosis of the jaw' OR 'bisphosphonate-associated mandibular osteonecrosis' OR 'bisphosphonate-associated necrosis of the maxilla' OR 'bisphosphonate-associated osteo-necrosis of the jaw' OR 'bisphosphonate-associated osteonecrosis of jaw' OR 'bisphosphonate-associated osteonecrosis of mandibular and maxillary bone' OR 'bisphosphonate-associated osteonecrosis of the jaw' OR 'bisphosphonate-associated osteonecrosis of the mandible' OR 'bisphosphonate-associated osteonecrosis of the mandibular bone' OR

'bisphosphonate-associated osteonecrosis of the maxilla' OR 'bisphosphonate-associated osteonecrosis of the maxillary bone' OR 'bisphosphonate-induced jaw osteonecrosis' OR 'bisphosphonate-induced mandible osteonecrosis' OR 'bisphosphonate-induced mandibular osteonecrosis' OR 'bisphosphonate-induced mandibular/maxillary osteonecrosis' OR 'bisphosphonate-induced maxillary osteonecrosis' OR 'bisphosphonate-induced osteonecrosis of the jaw' OR 'bisphosphonate-induced osteonecrosis of the mandible' OR 'bisphosphonate-induced osteonecrosis of the maxilla' OR 'bisphosphonate-related onj' OR 'bisphosphonate-related jaw osteonecrosis' OR 'bisphosphonate-related osteo-necrosis of the jaw' OR 'bisphosphonate-related osteonecrosis of jaw' OR 'bisphosphonate-related osteonecrosis of mandible' OR 'bisphosphonate-related osteonecrosis of the mandible' OR 'bisphosphonate-related osteonecrosis of the maxilla' OR 'bisphosphonateinduced osteonecrosis of the jaw' OR 'bisphosphonaterelated osteonecrosis of the jaw' OR 'bisphosphonates jaw osteonecrosis' OR 'bisphosphonates-induced osteonecrosis of the jaw' OR 'bisphosphonates-related osteonecrosis of jaw' OR 'bisphosphonates-related osteonecrosis of the jaw' OR 'jaw biphosphonate-osteonecrosis' OR 'mandible bisphosphonate-associated osteonecrosis' OR 'mandibular bisphosphonate-related osteonecrosis' OR 'zoledronic acid onj' OR 'zoledronic acid osteonecrosis of jaw' OR 'zoledronic acid osteonecrosis of the jaw' OR 'zoledronic acid-induced osteonecrosis of the jaw' OR 'zoledronic acid-related osteonecrosis of the jaw' OR 'medication related osteonecrosis of the jaw'/exp OR 'aonj' OR 'ar-onj' OR 'aronj' OR 'monj' OR 'mr-onj' OR 'mronj' OR 'antiresorptive agent-induced osteonecrosis of the jaw' OR 'antiresorptive agent-related onj' OR 'antiresorptive agent-related osteonecrosis of the jaw' OR 'antiresorptive-induced onj' OR 'antiresorptive-related onj' OR 'antiresorptive-related osteonecrosis of the jaw' OR 'drug-associated osteonecrosis of the jaw' OR 'drug-induced jaw osteonecrosis' OR 'drug-induced osteonecrosis of the jaw' OR 'drug-induced osteonecrosis of the mandible' OR 'drug-induced osteonecrosis of the maxilla' OR 'drug-related onj' OR 'drug-related jaw osteonecrosis' OR 'drug-related maxillary osteonecrosis' OR 'drug-related osteonecrosis of maxilla' OR 'drug-related osteonecrosis of the jaw' OR 'mandibular medication-related osteonecrosis' OR 'maxillary medication-related osteonecrosis' OR 'medication related osteonecrosis of the jaw' OR 'medication-associated jaw osteonecrosis' OR 'medication-associated osteonecrosis of the jaw' OR 'medication-associated osteonecrosis of the maxilla' OR 'medication-induced onj' OR 'medication-induced osteonecrosis of the jaw' OR 'medication-related onj' OR 'medication-related jaw osteonecrosis' OR 'medication-related osteonecrosis of jaw' OR 'medication-related osteonecrosis of the mandible' OR 'medication-related osteonecrosis of the maxilla')

Web of Science : 17

#1 saliva (All Fields) or **xerostomia** (All Fields) or **hyposalivation** (All Fields) or **"salivary gland"** (All Fields) or **sialadenitis** (All Fields) and **"Sjogren's syndrome"** (All Fields)

#2 "jaw osteonecrosis" (All Fields) or **"bisphosphonate related osteonecrosis of the jaw"** (All Fields) or **"medication related osteonecrosis of the jaw"** (All Fields)

#1 AND #2

Cochrane library : 2

"osteonecrosis of the jaw" OR "medication related osteonecrosis of the jaw" OR "bisphosphonate related osteonecrosis of the jaw" in All Text AND "xerostomia" OR "hyposalivation" in All Text
